# Open Fractures on the Field: Two Decades of Pediatric Sports Injuries in a Level 1 Trauma Cohort

**DOI:** 10.3390/jcm14186667

**Published:** 2025-09-22

**Authors:** Britta Chocholka, Lara Marie Bogensperger, Iryna Yegorova, Vanessa Groß, Manuela Jaindl, Bikash Parajuli, Sanika Rapole, Thomas Manfred Tiefenboeck, Stephan Payr

**Affiliations:** 1 University Clinic of Orthopedics and Trauma Surgery, Department of Trauma Surgery, Medical University of Vienna, 1090 Vienna, Austria; britta.chocholka@meduniwien.ac.at (B.C.); n11807478@students.meduniwien.ac.at (L.M.B.); iryna.yegorova@meduniwien.ac.at (I.Y.); n11916964@students.meduniwien.ac.at (V.G.); manuela.jaindl@meduniwien.ac.at (M.J.); thomas.tiefenboeck@meduniwien.ac.at (T.M.T.); 2 Section of Pediatric Trauma Surgery, Department of Trauma Surgery, University Clinic of Orthopedics and Trauma Surgery, Medical University of Vienna, 1090 Vienna, Austria; 3 Department of Orthopedics and Traumatology, Dhulikhel Hospital, Kathmandu University, Kavre 45200, Nepal; bikash@kusms.edu.np; 4 Department of Pediatric Orthopedics, Sancheti Institute for Orthopedics and Rehabilitation, Pune 411005, Maharashtra, India; drsanikarapole@gmail.com

**Keywords:** open fractures, pediatric trauma, sports-related, children, adolescents

## Abstract

**Background**: Open fractures in pediatric patients are uncommon but clinically relevant, often resulting from high-energy trauma or sports-related incidents. This study analyzes the demographic patterns, types of sports, injury mechanisms, treatment strategies, and outcomes in children and adolescents with sports-related open fractures. **Methods**: In this retrospective study, 74 pediatric patients with sports-related open fractures treated at a level 1 trauma center between 2002 and 2023 were documented. Parameters such as age, sex, fracture location, sport type, treatment modality, complications, and outcomes were evaluated. **Results**: The cohort included 74 patients, with a mean age of 13 ± 3.6 years. Open fractures of the upper extremity were most common (seen in 34 patients). Moreover, 10 open craniofacial and 27 open nasal fractures represented 50.0% of injuries, mainly in male athletes involved in contact sports. Soccer was the leading injury-related sport (n = 14; 18.9%). Surgical treatment was required in 28 patients (37.8%), most frequently using elastic stable intramedullary nailing, Kirschner wire fixation in the upper extremities or nasal bone reduction. Antibiotics were administered in 46 patients (62.2%), with a mean documented duration of 2.7 ± 3.1 days. An excellent outcome was documented in 95%. **Conclusions**: Sports-related open fractures in children primarily affect male adolescents in contact sports and involve the upper extremities and facial region. Conservative management is effective in stable, non-displaced and low-grade injuries. Surgical treatment is frequently indicated in open forearm fractures. The implementation of a structured trauma care protocol, incorporating early debridement, definitive treatment, and antibiotics, has been demonstrated to yield a safe and effective treatment outcome with a favorable prognosis for sports-related open fractures in children.

## 1. Introduction

Open fractures represent a clinically significant subset of pediatric trauma due to the combined involvement of bone and surrounding soft tissue structures. Although pediatric fractures are common, open injuries account for only 1–5% of all pediatric long bone fractures, making them a relatively rare but critical entity in trauma care [1,2,3]. These injuries are associated with an increased risk of infection, impaired fracture healing, growth disturbances, and long-term functional impairment if not managed promptly and appropriately [3,4]. The management of open fractures in children has evolved over recent decades, with increasing attention paid to the role of internal fixation, the choice between primary versus delayed wound closure, and the optimal duration of antibiotic prophylaxis [5,6,7]. The contemporary management since around 2014 has shifted from a rigid ‘6 h rule’ toward a paradigm emphasizing immediate intravenous antibiotics and timely debridement within 24 h, reserving emergent surgery for heavily contaminated or ischemic injuries. This change is supported by pediatric data showing no reduction in infection with debridement <6 h compared with <24 h when early antibiotics are administered, and by pathway studies demonstrating reduced door-to-antibiotic times [6,8,9].

Despite these challenges, children generally demonstrate a high capacity for healing, and with timely and evidence-based treatment, even open fractures can result in excellent clinical and functional outcomes [10,11,12].

Most existing studies on pediatric open fractures have concentrated on high-energy trauma, such as motor vehicle collisions, falls from height, or agricultural accidents [1,13]. These injury mechanisms are typically associated with severe fracture patterns and extensive soft tissue involvement [5,10]. In contrast, open fractures sustained during sports activities represent a different clinical and biomechanical context and remain considerably understudied in the pediatric trauma literature. Sports participation among children and adolescents is still a matter under investigation [14]; however, open fractures occurring in the context of organized or recreational sports have rarely been examined [15].

This lack of focused research is particularly notable given the unique characteristics of sports-related injuries in children and adolescents. Injury mechanisms vary considerably between contact and non-contact sports and often differ by sex, with boys and girls participating in different types of activities and being exposed to different biomechanical forces [13]. Moreover, clinical management strategies—including the need for surgical intervention, the use of antibiotics, and the timing of wound closure—may differ from those applied in high-energy trauma, yet evidence-based guidance in this specific context remains limited. Given the underrepresentation of data on sports-related open fractures in children and adolescents in the current literature, the present study aims to provide a detailed epidemiological and clinical overview. The objective of this study is to provide a comprehensive 20-year retrospective analysis of pediatric open fractures sustained during sports activities at a level 1 trauma center, identifying sport- and sex-specific injury patterns, injured regions, treatment modalities and clinical outcomes.

## 2. Materials and Methods

This retrospective study was approved by the Ethics Committee of the Medical University of Vienna (EC-Code: 2075/2023) on 26 February 2025 and conducted according to the Declaration of Helsinki and its latest amendments.

Patients younger than 18 years who sustained sports-related open fractures between January 2002 and December 2023 and were treated at the level 1 trauma center at the Department of Trauma Surgery at the Medical University of Vienna were included. Eligible cases were identified through internal hospital databases and electronic medical records. The inclusion criteria comprised the presence of at least one documented open fracture, sustained during either organized or recreational sports activity. Patients with fractures unrelated to sports, incomplete documentation (3 patients because of early transfer), or pathological fracture etiologies were excluded.

The extracted parameters included demographic information (age, sex), injury characteristics (fracture location and type, laterality), type of sport, treatment approach (surgical vs. non-surgical), antibiotic administration and duration, complications, and clinical outcome. Patients were also categorized according to age into children (0–10 years) and adolescents (11–17 years). Sports were classified into contact and non-contact categories. Injury mechanisms were analyzed based on the distinction between organized and recreational sports. Organized sports were defined as structured team or individual activities typically associated with clubs or schools, while recreational sports included informal or leisure-based activities without formal supervision. The clinical outcome was described by whether patients regained full functionality without restrictions in mobility, sensation or participation in daily or sporting activities. Descriptive analysis, including mean ± standard deviation (SD) and range, was performed for the entire patient cohort. In order to provide an epidemiological overview, the following parameters were included: age in years, sex, injury mechanism, diagnosis, treatment (conservative or surgical), hospital stay in days, follow-up (FUP) defined in days between presentation and last documented visit/day at our department and deficits at the last FUP.

For metric variables (age or FUP), mean values and standard deviation values, and for categorial variables (injury mechanism, injuries and type of treatment), frequencies and percentages, were determined.

All statistical analyses were performed using Microsoft^®^ Excel macOS software (Version 16.42 Microsoft Corp., Redmond, WA, USA) and SPSS^®^ software (Version 27.0.0., SPSS Inc.: Chicago, IL, USA).

## 3. Results

A total of 74 pediatric patients (male: 50, 67.6%; female: 24, 32.4%) sustained sports-related open fractures. The overall mean age was 13 ± 3.6 years (range: 5–18 years). Male patients had a mean age of 13.4 ± 3.6 years (range: 5–18), while female patients had a mean age of 12.4 ± 3.7 years (range: 5–18). The majority of patients were adolescents aged 10 to 18 years, accounting for 55 cases (74.3%).

### 3.1. Injury Mechanism/Type of Sport

Across all 74 patients, falls were the predominant injury mechanism (42%), followed by collisions (16%) and unknown circumstances (18%). Entrapment (7%) and direct blows, with or without sporting equipment (10%), were less frequent, while object-related injuries (ball, puck, or stick) and kicks were rare (<5%). When stratified by age, distinct patterns emerged: in children (0–10 years; n = 19), falls accounted for one-third of injuries (32%), while entrapment (21%) and collisions (16%) were also common.

In contrast, adolescents (11–18 years; n = 55) most frequently sustained injuries through falls (45%), followed by collisions (16%) and direct blows (9%). Notably, object-related mechanisms were exclusively observed in the adolescent group (7%).

Organized sports accounted for a substantial proportion of cases (Table 1), particularly in contact disciplines. Soccer represented the leading sport (n = 14; all male; mean age 14.4 ± 3.4 years), with injuries most frequently resulting from collisions (n = 5) and falls (n = 4) but also including direct blows (n = 2), one twisting injury, and two cases with unknown mechanisms. Horseback riding (n = 7; six females, one male; mean age 12.4 ± 2.4 years) injuries were predominantly caused by falls (n = 5), along with one collision and one kick. Ice skating (n = 5; four males, one female; mean age 11.8 ± 4.1 years) was associated with three falls and two cases of unknown circumstances. Handball (n = 4; one male, three female) involved two direct blows and two unknown mechanisms, while volleyball (n = 4; two male, two female) included two collisions and two injuries of unknown cause.

In ice hockey (n = 2; all male) and field hockey (one male), the cases were due to stick-related impacts (n = 2), with one additional case following a puck strike.

Recreational activities also contributed substantially to the overall injury burden (Table 2). Skateboarding was the most frequent activity (n = 6; five males, one female; mean age 15.0 ± 2.4 years), with injuries typically sustained through falls (n = 6). Cycling ranked second (n = 5; three males, two females; mean age 7.6 ± 5.3 years), predominantly affecting younger children who fell during unsupervised play. Water sports (n = 4; three males, one female; mean age 12.5 ± 1.7 years) and casual fitness training (n = 3; two males, one female; mean age 16.7 ± 1.2 years) were also represented, most often associated with falls or equipment-related trauma.

### 3.2. Region of Injury

#### 3.2.1. Skull and Face

A total of 37 patients (50.0%) sustained open injuries involving the skull or facial region. Among these, 27 were isolated open nasal bone fractures, representing the most frequent type of craniofacial trauma. The remaining ten cases included six open skull base fractures, four mandibular fractures and one orbital wall fracture, which was combined with one open skull base fracture. All injuries were classified as open due to mucosal disruption or soft tissue laceration. The mean age of all the patients with skull or facial fractures was 14.3 ± 3.0 years (range 7 to 18 years). Of the 37 patients, 23 were male (62.2%) and 14 female (37.8%). Injuries in male patients occurred most frequently during contact or equipment-based sports such as soccer (n = 6), basketball (n = 2), handball (n = 1), and ice hockey (n = 2), as well as during recreational winter activities, including ice skating (n = 3) and sledding (n = 1). The typical mechanisms included elbow-to-face impacts, head-to-head collisions, or unprotected falls. Female patients sustained craniofacial injuries predominantly during falls from bicycles (n = 2), scooters (n = 2), or horses (n = 2). One additional case occurred during a collision while playing volleyball (n = 1). Among the patients with open skull base fractures, one case (15-year-old male) required neurosurgical intervention for an epidural hematoma, while no other intracranial complications were observed. Among the 27 open nasal bone fractures, 6 (22.2%) required surgical reduction under general anesthesia, whereas 21 (77.8%) were treated conservatively. Craniofacial injuries occurred predominantly in adolescents (n = 31; 83.8%), whereas only a minority were observed in children aged 0–11 years (n = 6; 16.2%).

#### 3.2.2. Extremities

Open fractures of the extremities were documented in 37 of the 74 patients (50.0%). A total of 25 (67.6%) were male (mean age: 12.2 ± 3.8 years) and 12 (32.4%) were female (mean age: 11.8 ± 3.9 years).

Fractures of the upper extremity were significantly more common than lower limb injuries. The most frequently affected region was the hand and fingers (n = 19), followed by the forearm (radius and/or ulna; n = 12), supracondylar humerus (n = 2) and metacarpal region (n = 1). Lower extremity involvement was limited to two tibial fractures and the metatarsal bone (n = 1).

Among the 16 long bone fractures (12 forearm, two lower leg, and two supracondylar humerus fractures), the majority were classified as Gustilo–Anderson grade I (13/16; 81.3%). Grade II injuries accounted for two cases (12.5%), while grade III was observed in one case (6.3%). Of the 37 extremity fractures, 33 involved the upper limb and were almost evenly distributed between children (16/37; 43.2%) and adolescents (17/37; 45.9%), whereas all the lower limb fractures (4/37; 10.8%) occurred exclusively in adolescents.

Open fractures of the extremities typically resulted from direct trauma, including high velocity ball contact, in sports such as soccer and handball or direct falls additionally, including skateboarding and ice skating. Finger and hand injuries were particularly common in ball sports, while forearm fractures most often occurred in younger children attempting to break their fall with outstretched arms.

### 3.3. Treatment Plus Antibiotic Use

A total of 28/74 pediatric patients (37.8%) underwent surgical treatment (mean age: 12.5 ± 3 years) (22 extremity fractures and six nasal bone fractures), while 46 patients (62.2%) were treated conservatively (mean age: 13.5 ± 4 years) (15 extremity fractures and 31 craniofacial fractures) (Table 3). The treatment modality was chosen based on the fracture stability, soft tissue condition, and patient age. The surgical procedures were performed after the ensured fasting of the patients (<6 h), and antibiotic treatment for fractures of the extremities was administered at the time of diagnosis.

The most frequently performed procedures were Kirschner wire fixation (n = 8; mean age: 12.8 ± 3.4 years), nasal bone reduction (n = 6; mean age: 13.7 ± 2.4 years) and plate osteosynthesis (n = 4; mean age: 13.0 ± 1.4 years). Elastic stable intramedullary nailing (ESIN) was applied in seven cases (mean age: 11.4 ± 2.4 years), primarily for diaphyseal fractures of the forearm. Isolated cases were treated with external fixation (n = 1; female; 15.0 years), screw fixation (n = 1; female; 16.0 years), and bone reconstruction (n = 1; male; 10.0 years), reflecting more complex fracture morphologies.

The conservative treatment of 46 patients (62.2%) included immobilization techniques such as finger splints and bandages, forearm and below knee cast as well as soft bandages and taping. Conservative treatment was particularly common in uncomplicated open injuries affecting the fingers and nasal bone. Overall, 12 patients with open finger fractures, two patients with forearm fractures, one patient with open a metatarsal bone fracture and 31 patients with open craniofacial fractures were treated conservatively.

Overall, antibiotic prophylaxis was administered in 49/74 patients (66.2%), including both surgical and conservative cases (Table 4). According to the injured regions, 27 of the 37 extremity fractures and 22 of the 37 craniofacial fractures received antibiotic treatment. In 49 patients with recorded antibiotic duration, the mean treatment length was 2.7 ± 3.1 days.

Surgical treatment was slightly more frequent in adolescents (22/55; 40.0%) compared to children (6/19; 31.6%), whereas conservative management remained common in both age groups. Antibiotics were administered with comparable proportions in children (60.9%) and adolescents (62.7%). The mean duration of antibiotic therapy was 5.8 ± 1.5 days (range 1–8) and relatively the same in both age groups.

### 3.4. Temporal Trends in Treatment and Antibiotic Use (2002–2012 vs. 2013–2023)

When stratified by time period, the distribution of fracture sites revealed notable differences, with 28 extremity fractures, 16 nasal bone fractures, and seven other craniofacial fractures documented between 2002 and 2012, compared to nine extremity fractures, 11 nasal bone fractures, and three other craniofacial fractures in 2013–2023.

Surgical treatment was relatively similar in both periods, with K-wires and elastic stable intramedullary nails being most commonly used for fractures of long bones. The three cases of nasal bone reductions remained constant in both periods, while other craniofacial fractures were treated surgically more frequently in the later decade.

The administration of antibiotics yielded the following results. For fractures of the extremities, the proportion of patients receiving antibiotics was comparable, at 80% in 2002–2012 and 77.8% in 2013–2023, with the average duration increasing from 2.5 ± 3.0 to 5.3 ± 2.3 days. In cases of nasal bone fractures, antibiotics were used in 7/16 cases compared to 7/11 cases, with a longer treatment duration.

## 4. Complications

Post-traumatic complications requiring revision surgery were documented in two patients. The first case involved a 15-year-old male patient with an open mandibular fracture that was operatively treated with plating on the day of injury. Four days later, revision surgery became necessary due to fragment tilting at the ramus mandibulae and medial dislocation of the condyle. Approximately six months after the initial trauma, alveolar ridge atrophy developed, necessitating reconstruction with an iliac crest bone graft. At the one-year follow-up, the patient was largely asymptomatic, presenting only with a widened submandibular scar and a slightly reduced alveolar ridge height. The second case was a 12-year-old female who sustained a bilateral open mandibular fracture following a fall from a horse. Initial fixation was performed on the day of trauma. Four months later, early plate removal was required due to exposure, followed by three additional procedures for soft tissue reconstruction in the context of persistent intraoral wound healing disorder and subsequent scar correction. During the final operation, resection of an exostosis at the medial tibial condyle of the left knee, which had developed from a previous injury, was also performed.

## 5. Outcome

The clinical outcome was available for all 74 patients at the final follow-up (mean FUP: 56 ± 99.5 days). A total of 70 patients (94.6%) achieved full functional recovery, without limitations in mobility, sensation, or participation in daily or athletic activities.

Four documented impairments were observed in this cohort. One case involved a 14-year-old male with an open metacarpal fracture who presented with mild residual hyposensitivity around the scar area. A 17-year-old male with a skull base fracture reported persistent occipital headaches and dizziness, although no intracerebral hemorrhage was detected during the initial work-up. Another case concerned a 13-year-old male with an open forearm fracture who developed muscular atrophy and ulnar nerve hypesthesia. Finally, a 9-year-old female with a supracondylar humerus fracture showed a residual extension deficit of 5° at the elbow, though overall function was preserved. None of these impairments interfered with the patients’ daily or athletic activities.

The clinical outcomes were comparable across age groups, with full functional recovery achieved in 18 of 19 children (95.7%) and 52 of 55 adolescents (96.1%).

## 6. Discussion

This retrospective analysis of 74 pediatric patients with open fractures sustained during sports activities over a 20-year period at a certified level 1 trauma center shows that sports-related open fractures in children and adolescents are extremely rare yet exhibit specific patterns regarding sex, type of sport, and anatomical localization, therefore presenting a very distinct epidemiological database on pediatric trauma from what is available in the current literature. The findings also show a predominance of male patients, who accounted for almost 70% of all patients in this cohort, which is consistent with previous studies mentioning this overrepresentation in sports-related trauma, particularly in high-risk contact sports such as soccer, martial arts, or skateboarding [2,15,16,17,18].

In this study, soccer was the most common sport, associated with open fractures in almost 20% of cases. These soccer injuries involved the upper extremities (radius, ulna and fingers) or the nasal bone and were caused by falls, collisions, or a direct impact with the ball. All the soccer-related injuries were exclusively seen in male patients. These findings align with prior reports that also identify the upper limb and the nasal area as the most frequently injured anatomical region in youth soccer, where falls and physical contact are frequent [19,20]. While nasal bone fractures are often considered minor, their classification as open fractures—due to mucosal disruption and exposure to nasal flora—raises questions about appropriate management. In this presented cohort, no postoperative infections were observed, regardless of whether antibiotic prophylaxis was administered or not, supporting the recent literature suggesting that routine antibiotics may not be necessary in uncomplicated nasal bone fractures [21,22,23]. The present data further suggest that open nasal bone fractures with small, clean lacerations (<1 cm) may achieve favorable clinical outcomes even in the absence of prophylactic antibiotics. In this cohort, all the patients recovered without infection or functional impairment, irrespective of antibiotic use. This observation supports the notion that, in carefully selected low-grade injuries with minimal contamination, the benefit of routine antibiotic prophylaxis may be limited. While larger prospective studies are needed to validate this finding, it highlights the potential to reduce unnecessary antibiotic exposure in pediatric patients with minor open nasal fractures.

With regard to fracture severity, in this study, the open extremity fractures of the long bones classified as Gustilo–Anderson were 13 (81.3%) type I, two (12.5%) type II and only one (6.3%) type III. This distribution is consistent with prior studies reporting that over 80% of pediatric open fractures related to sports are of low to moderate severity [2,24]. The administered antibiotic single-shot or short-term regimens in this study reflect current pediatric recommendations advocating limited prophylaxis in clean open fractures, particularly when early debridement and definitive wound care are achieved [21,22,25]. Importantly, primary wound closure and definitive fracture fixation was achieved in more than 85% (n = 62) of cases, with excellent clinical outcomes in 95% of patients. Historically, pediatric open fractures were managed cautiously, and delayed closure was often advocated to minimize the risk of infection, consistent with the former ‘6 h rule’ for debridement and prolonged antibiotic regimens. However, more recent evidence and international guidelines [8,9,26] have shifted toward a paradigm emphasizing immediate intravenous antibiotics, timely wound excision within 12–24 h, and early definitive fixation with soft tissue coverage ideally within 72 h. Several studies have further demonstrated that primary closure in Gustilo–Anderson grade I and II fractures is both safe and effective when combined with adequate debridement and antibiotic prophylaxis, resulting in low complication and infection rates [5,7]. The authors’ results align with contemporary recommendations, showing low complication rates (5%) and no cases of deep infection or nonunion, thereby underscoring the safety of early primary closure in the pediatric trauma population. Although treatment guidelines have undergone a paradigm shift during the last two decades, this did not substantially affect the data, as all the patients in both study periods were treated surgically on the day of trauma, usually within 6 h after admission. Thus, the changes in international recommendations mainly concerned the conceptual framework of acute management but had little impact on the authors’ clinical practice. This consistency renders the results across both decades directly comparable. While one might argue that a 20-year observation period could limit comparability, the setting of a certified level 1 trauma center with standardized acute care ensured uniform management and reliable outcome assessment throughout the entire study period.

Another important aspect of this study is the temporal trend in injury incidence. Over the 20-year observation period, we noted a slight but consistent decline in the annual number of open fractures related to sports. While our dataset is limited to a single center, this observation aligns with broader epidemiological findings suggesting a reduction in severe pediatric sports injuries over the last decade [14,15]. Several factors may contribute to this trend, including improved awareness of injury mechanisms, stricter safety guidelines in organized youth sports, and the increasing use of protective equipment. For example, the widespread adoption of shin guards and face protection in American football and the mandatory use of helmets and safety vests in equestrian sports have likely contributed to the observed reduction in high-energy and open injuries [27,28,29]. Furthermore, improved coaching standards and better supervision in recreational and competitive sports environments may also play a role in injury prevention [30]. The authors posit that the training of coaches, encompassing the formulation of training plans and programs, is of paramount importance, particularly in sports that demand a high level of technical proficiency, such as contact sports, as well as gymnastics, weightlifting, ice skating and horse riding. It is imperative that contact sports and team sports incorporate specialized training for referees, in addition to the implementation of stringent regulations pertaining to foul play, which have the potential to contribute to the prevention of such incidents. The following essay will provide a comprehensive overview of the relevant literature on the subject. It is reasonable to assume that the aforementioned points will prove more relevant in the context of amateur sports than professional sports.

A limitation of this study is definitely the respective study design, which is consistent with a certain loss of data. Incomplete data include the use of protection gear and helmets, which is unfortunate for such a specific trauma population.

Despite its retrospective nature, the strength of this study is its long observation period, presenting a large sample size and comprehensive epidemiological data on relatively rare and specific injuries in children and adolescents that are hardly represented in the current literature. Furthermore, this study provides a complete compilation of exclusively open fractures in children and adolescents only and is not limited to a specific type of fracture, as is common in the literature. In addition, the findings not only confirm previously described injury patterns but also underscore the effectiveness of early antibiotic coverage, debridement and definitive surgical care of open fractures, even in the context of sports trauma, leading to excellent long-term function when performed within a structured clinical framework.

## 7. Conclusions

Sports-related open fractures in children and adolescents represent a distinct injury pattern with clear gender- and sport-specific characteristics. Male recreational athletes are particularly affected in contact sports such as soccer, with frequent injuries to the upper limbs and skull, while girls are more often at risk in equestrian sports. Conservative management is effective in stable, non-displaced, and low-grade injuries, whereas surgical treatment is frequently required in open forearm fractures. Overall, most injuries are minor to moderate in severity and can be treated safely and effectively with early debridement, definitive stabilization, and appropriate antibiotic coverage. Importantly, the present findings also suggest that small, clean open nasal bone fractures may achieve excellent outcomes even without routine antibiotic prophylaxis, underscoring the potential for a more selective approach to antibiotic use in pediatric trauma care.

## Figures and Tables

**Table 1:** Organized sports.

Sports	n	Percentage	Male	Female	Mean Age ± SD
Soccer	14	29.2%	14	0	14.4 ± 3.4
Horseback riding	7	14.6%	1	6	12.4 ± 2.4
Ice skating	5	10.4%	4	1	11.8 ± 4.1
Handball	4	8.2%	1	3	17.0 ± 1.0
Volleyball	4	8.2%	2	2	13.0 ± 2.0
Basketball	3	6.3%	2	1	17.5 ± 0.5
Gymnastics	2	4.2%	0	2	11.3 ± 1.7
Ice hockey	2	4.2%	2	0	13.2 ± 2.6
Martial arts	2	4.2%	2	0	13.0 ± 0.0
Tennis	2	4.2%	1	1	11.5 ± 1.7
Field hockey	1	2.1%	1	0	13.0 ± 0.0
Wrestling	1	2.1%	1	0	13.0 ± 0.0
American football	1	2.1%	1	0	14.0 ± 0.0
Total	48	100%	32	16	13.5 ± 1.8

**Table 2:** Recreational activities.

Activities	n	Percentage	Male	Female	Mean Age ± SD
Skateboarding	6	23.1%	5	1	15.0 ± 2.4
Cycling	5	19.2%	3	2	7.6 ± 5.3
Water spots	4	15.5%	3	1	12.5 ± 1.7
Casual fitness	3	11.5%	2	1	16.7 ± 1.2
Bowling	3	11.5%	2	1	7.7 ± 2.1
Inline skating	2	7.7%	1	1	14.5 ± 2.5
Sledding	2	7.7%	1	1	7.0 ± 1.0
Trampolining	1	3.8%	1	0	10.0 ± 0.0
Total	26	100%	18	8	11.4 ± 3.6

**Table 3:** Treatment modality.

	Method	n	Percentage	Male	Female	Mean Age ± SD
Surgical	K-Wire	8	10.8%	7	1	12.8 ± 3.4
	ESIN	7	9.4%	5	2	11.4 ± 2.4
	Nasal Reduction	6	8.1%	4	2	13.7 ± 2.4
	Plate Osteosynthesis	4	5.4%	1	3	13.0 ± 1.4
	External Fixation	1	1.4%	1	0	15.0 ± 0.0
	Reconstruction	1	1.4%	1	0	10.0 ± 0.0
	Screw Fixation	1	1.4%	1	0	16.0 ± 0.0
Conservative	No Device	31	41.8%	21	10	14.3 ± 3.5
	Finger Splint	8	10.7%	5	3	12.3 ± 4.0
	Finger Bandage	3	4.1%	1	2	7.0 ± 1.0
	Forearm Cast	2	2.7%	1	1	9.5 ± 6.4
	Taping	1	1.4%	1	0	13.0 ± 0.0
	Below Knee Cast	1	1.4%	1	0	12.0 ± 0.0
Total		74	100%	50	24	13.0 ± 3.7

**Table 4:** Administration of antibiotics.

		Treatment	Antibiotics	No Antibiotics	Total
Diagnosis	Finger fracture	Conservative	11	4	15
		Surgical	4	-	4
	Mandibular fracture	Surgical	2	2	4
	Metatarsal fracture	Conservative	1	-	1
	Metacarpal fracture	Conservative	1	-	1
	Nasal bone fracture	Conservative	8	13	21
		Surgical	6	-	6
	Skull base fracture	Conservative	5	-	5
		Surgical	1	-	1
	Supracondylar fracture	Surgical	2	-	2
	Forearm fracture	Conservative	1	6	7
		Surgical	5	-	5
	Lower leg fracture	Surgical	2	-	2
Total			49	25	74

## Data Availability

The datasets generated and/or analyzed in the current study are not publicly available due to data privacy but are available from the corresponding author on reasonable request.
